# Stromal cell derived factor-1: its influence on invasiveness and migration of breast cancer cells *in vitro*, and its association with prognosis and survival in human breast cancer

**DOI:** 10.1186/bcr1022

**Published:** 2005-04-04

**Authors:** Hua Kang, Gareth Watkins, Christian Parr, Anthony Douglas-Jones, Robert E Mansel, Wen G Jiang

**Affiliations:** 1 Metastasis and Angiogenesis Research Group, Wales College of Medicine, Cardiff University, Cardiff, UK; 2 Currently working in the Department of Surgery, Xuanwu Hospital, Beijing, China; 3 Department of Pathology, Wales College of Medicine, Cardiff University, Cardiff, UK

## Abstract

**Introduction:**

Stromal cell-derived factor (SDF)-1 (CXC chemokine ligand-12) is a member of the CXC subfamily of chemokines, which, through its cognate receptor (CXC chemokine receptor [CXCR]4), plays an important role in chemotaxis of cancer cells and in tumour metastasis. We conducted the present study to evaluate the effect of SDF-1 on the invasiveness and migration of breast cancer cells, and we analyzed the expression of SDF-1 and its relation to clinicopathological features and clinical outcomes in human breast cancer.

**Method:**

Expression of SDF-1 mRNA in breast cancer, endothelial (HECV) and fibroblast (MRC5) cell lines and in human breast tissues were studied using RT-PCR. MDA-MB-231 cells were transfected with a SDF-1 expression vector, and their invasiveness and migration was tested *in vitro*. In addition, the expression of SDF-1 was investigated using immunohistochemistry and quantitative RT-PCR in samples of normal human mammary tissue (*n *= 32) and mammary tumour (*n *= 120).

**Results:**

SDF-1 expression was identified in MRC5, MDA-MB-435s and MDA-MB-436 cell lines, but CXCR4 expression was detected in all cell lines and breast tissues. An autocrine loop was created following transfection of MDA-MB-231 (which was CXCR4 positive and SDF-1 negative) with a mammalian expression cassette encoding SDF-1 (MDA-MB-231SDF1^+/+^) or with control plasmid pcDNA4/GFP (MDA-MB-231^+/-^). MDA-MB-231SDF1^+/+ ^cells exhibited significantly greater invasion and migration potential (in transfected cells versus in wild type and empty MDA-MB-231^+/-^; *P *< 0.01). In mammary tissues SDF-1 staining was primarily seen in stromal cells and weakly in mammary epithelial cells. Significantly higher levels of SDF-1 were seen in node-positive than in node-negative tumours (*P *= 0.05), in tumours that metastasized (*P *= 0.05), and tumours from patients who died (*P *= 0.03) than in tumours from patients who were disease free. It was most notable that levels of SDF-1 correlated significantly with overall survival (*P *= 0.001) and incidence-free survival (*P *= 0.035).

**Conclusion:**

SDF-1 can increase the invasiveness and migration of breast cancer cells. Its levels correlated with node involvement and long-term survival in patients with breast cancer. SDF-1 may therefore have potential value in assessing clinical outcomes of patients with breast cancer.

## Introduction

Breast cancer is the most common female cancer in the UK and USA. One in ten women will develop breast cancer in their lifetime in Western countries [1,2]. The poor prognosis of patients with breast cancer is related to tumour recurrence and metastasis [3,4]. Breast cancer is characterized by metastasis to regional lymph nodes, bone marrow, lungs and the liver [5]. Previous studies [6,7] demonstrated that sites of metastasis are determined not only by the characteristics of neoplastic cells but also by the microenvironment of the specific organs. Organ specific attractant molecules can promote homing of tumour cells to particular sites [5,7].

Stromal cell-derived factor (SDF)-1 (CXC chemokine ligand-12) is a member of CXC chemokine family, which was initially cloned from murine bone marrow and characterized as a pre-B-cell growth stimulating factor [8-10]. SDF-1 exerts effects through its cognate receptor CXC chemokine receptor (CXCR4), which is the only physiological receptor for SDF-1 and is known to play roles in chemotaxis [11,12], haematopoiesis [13,14], vasculogenesis [15-17] and tumour spread and metastasis [6,18,19]. It was recently shown that CXCR4 is involved in homing of tumour cells to specific organs and in tumour progression [6,18-20]. Muller and coworkers [19] found that SDF-1/CXCR4 plays a critical role in determining the metastatic destination of breast cancer cells. Moreover, they demonstrated that neutralization with a specific monoclonal antibody against CXCR4 effectively inhibited the metastasis of breast cancer cells to the lung or lymph nodes in mice [19].

However, despite the accumulated information on CXCR4, few studies have been conducted to evaluate SDF-1 expression and its prognostic value in patients with breast cancer. In the present study we evaluated the effect of the SDF-1 gene in breast cancer cells on their invasive and migration properties, using a SDF-1 transfection technique. Furthermore, we analyzed SDF-1 expression by real-time quantitative RT-PCR and immunohistochemical staining, and its relation with clinicopathological features and clinical outcomes in human breast cancer.

## Materials and method

### Materials

The RNA extraction kit and reverse transcription kit were obtained from AbGene Ltd (Epsom, Surrey, UK). PCR primers were designed using Beacon Designer (Palo Alto, CA, USA) and synthesized by Invitrogen Ltd (Paisley, UK). Molecular biology grade agarose and DNA ladder were obtained from Invitrogen. The master mix for routine PCR and quantitative PCR was from AbGene Ltd. Goat anti-human SDF-1 polyclonal antibodies and rabbit anti-human CXCR4 polyclonal antibody were purchased from Santa Cruz Biotechnology Ltd (Santa Cruz, CA, USA). Peroxidase conjugated anti-goat and anti-rabbit antibodies were obtained from Sigma (Poole, Dorset, England, UK) and a biotin universal staining kit was from Vector Laboratories (Nottingham, England, UK). Matrigel (reconstituted basement membrane) was purchased from Collaborative Research Products (Bedford, MA, USA). A transwell plate equipped with a porous insert (pore size 8 μm] was obtained from Becton Dickinson Labware (Oxford, UK).

### Cell lines and culture conditions

The following human breast cancer cell lines were used: MDA-MB-157, MDA-MB-231, MDA-MB-435s, MDA-MB-436, MDA-MB-453, MCF7, BT549 and ZR751 (purchased from the European Collection of Animal Cell Cultures, Salisbury, UK). Human foetal lung fibroblast cell line MRC5 (from the European Collection of Animal Cell Cultures) and human vascular endothelial cell line HECV (from the Biology and Cellular and Molecular Pathology Department, Naples, Italy) were also used. The cell lines were maintained in Dulbecco's modified Eagle's medium with 10% foetal calf serum, 100 units/ml penicillin and 100 μg/ml streptomycin, and at 37°C in a humid atmosphere of 5% carbon dioxide/95% air.

### Construction of SDF-1 expression cassette

Full-length human SDF-1 cDNA was obtained by amplifying the mRNA from normal human fibroblasts, using RT-PCR with the following primers: sdf1exf1 (5'-atgaacgccaaggtcgtg-3'] and SDF1ExR1 (5'-tcacatcttgaacctcttgtt-3'). The discrete SDF-1 product was subsequently TA cloned into pcDNA4/GFP-NT vector (Invitrogen Ltd), followed by transformation using One-Shot *E. coli *(Invitrogen Ltd), verification, and amplification. Purified plasmid, or control plasmid, was used to transfect MDA-MB-231 cells by electroporation using an electroporator, EasyJet Plus (Flowgen, Boughton, Kent, England, UK), followed by selection with G418 (Sigma). Stable SDF-1 transfectant (MDA-MB-231SDF1^+/+^), or stable control plasmid transfectant (MDA-MB-231^+/-^), was subsequently established and verified.

### *In vitro *invasion analysis

This technique was previously reported and modified in our laboratory [21]. Briefly, transwell inserts with 8 μm pore size were coated with 50 μg Matrigel and dried, before being rehydrated. Breast cancer cells (20 × 10^3^) were added to each well. After 96 hours cells that had migrated through the matrix and stuck to the other side of the insert were fixed (4% formalin), stained with 0.5% (weight/volume) crystal violet and counted under a microscope.

### Migration assay

The migration assay was based on a method established in our laboratories [22]. Confluent cells were first overlaid with light mineral oil and then placed on a stage heated to 37°C. The cell monolayer was scratched using a fine plastic pipette, creating wounds of approximate 250 μm in width. These wounds were then continuously monitored using a digital camera and time-lapse video recorder. Images were subsequently obtained at 10-min intervals and analyzed using a motion analysis package (Optimas 6) (Optimas Corporation, Bothell, Washington, USA). The accumulated distance that cells travelled over a period of 10 min was analyzed. More than 20 cells were analyzed in each setting, and data were automatically processed using Excel software.

### Tissue samples

Tissue samples were collected from patients with breast cancer who had undergone mastectomy. Breast cancer tissue samples (*n *= 120) and normal mammary tissue samples (from the same patients but away from tumours, and free from tumour cells, as confirmed by subsequent histological analysis; *n *= 32) were collected immediately after surgery and stored at -80°C until use. Patients were routinely followed clinically after surgery and details were stored in a database. The median follow-up period was 72 months. Details of histology were obtained from pathology reports (Table 1).

### RT-PCR and real-time Quantitative PCR

Frozen sections of tissues were cut at a thickness of 5–10 μm and kept for immunohistochemistry and routine histology. An additional 15–20 sections were mixed and homogenized using a hand-held homogenizer, in ice-cold RNA extraction solution. Total RNA extraction from frozen tissues and culture cells was performed using standard RNA isolation kit. The concentration of RNA was determined using an ultraviolet spectrophotometer. Reverse transcription was conducted using a reverse transcription kit with an anchored oligo [dT] primer supplied by AbGene Ltd, using 1 μg total RNA in a 96-well plate. The quality of cDNA was verified using β-actin primers (5'-caggaggttgaaggactaaa-3' and 5'-gggatcagttttctttgtca-3').

Conventional PCR was performed with specific primers for SDF-1 and CXCR4. Amplication conditions were as follows: 94°C for 5 min, followed by 40 cycles of 94°C for 30 s, 55°C for 1 min and 72°C for 1 min. This was followed by a final extension for 5 min at 72°C. The products were visualized on 2% agarose gel after stain with ethidium brominde.

The level of SDF-1 and CXCR4 transcripts from the prepared cDNA was determined using a real-time quantitative PCR, based on Amplifluor technology, modified from a method reported previously [23]. (TCS Biologicals Oxford, England, UK) Briefly, pairs of PCR primers were similarly designed using Beacon Designer software, version 2 (Biosoft International, Palo Alto, California, USA) (primer sequence: sense SDF-1 5'-ttcaggagtacctggagaaa-3', CXCR4 5'-cttcttaactggcattgtgg-3'; antisense SDF-1 5'-actgaacctgaccgtacacctaacactggt-3', CXCR4 5'-actgaacctgaccgtacagtgatgacaaag-3'), but an additional sequence was added to one of the primers [24]. This is known as the Z sequence (5'-actgaacctgaccgtaca-3') which is complementary to the universal Z probe (Intergen Inc, Oxford, UK). The primers used for quantitation of oestrogen receptor (ER) and ER-β were as we reported previously [23] (ER; 5'-cctactacctggagaacgag-3' and 5'-ctcttcggtcttttcgtatg-3'; and ER-β: 5'-aaaagaatcattcaatgaca-3' and 5'-attaacacctccatccaaca-3'). Primers used to quantify CK19 were as previously reported (5'-caggtccgaggttactgac-3' and 5'-actgaacctgaccgtacacactttctgc cagtgtgtcttc-3', respectively) [23,25].

The reaction was carried out using the following: Hot-start Q-master mix (AbGene Ltd); 10 pmol of specific forward primer; 1 pmol reverse primer, which has the Z sequence; 10 pmol of FAMtagged probe (Intergen Inc), and cDNA from ~50 ng of RNA. The reaction was conducted using IcyclerIQ (Bio-Rad, Hemel Hempstead, Herts, England, UK), which is equipped with an optic unit that allows real-time detection of 96 reactions, under the following conditions: 94°C for 12 min and 50 cycles of 94°C for 15 s, 55°C for 40 s, and 72°C for 20 s [15]. The levels of transcripts were generated from a standard that was simultaneously amplified with the samples.

### Immunohistochemical staining of SDF-1 proteins

In the present study, normal breast tissue samples (*n *= 32) and their respective matched breast tumour samples (*n *= 32) were used for immunohistochmemical analysis. Tissues were frozen and sectioned at a thickness of 6 μm using a cryostat. The sections were mounted on SuperFrostPlus microscope slides (Ramond A Lamb, London, England, UK) and were air-dried and then fixed in a mixture of 50% acetone and 50% methanol. The sections were then placed in Optimax wash buffer (San Ramon, California, USA) for 5–10 min to rehydrate. Sections were incubated for 20 min in a 0.6% bovine serum albumin blocking solution and were then probed with the primary antibody for 1 hour. After extensive washings in buffer, sections were incubated for 30 min in the secondary biotinylated antibody (Multilink Swine anti-goat and anti-rabbit immunoglobulin; Dako Inc., Angel Drove, Ely, Cambridgeshire, England, UK). After washing, avidin biotin complex (Vector Laboratories) was then applied to the sections followed once more by extensive washings. Diaminobenzidine chromogen (Vector Laboratories) was then added to the sections, which were then incubated in the dark for 5 min. Sections were then counterstained in Gill's haematoxylin and were dehydrated in ascending grades of methanol before clearing in xylene and mounting under a coverslip.

### Statistical analysis

Statistical analysis was carried out using the Mann–Whitney U-test and the Kruskal–Wallis test, survival analysis was using Kaplan–Meier survival analysis and Cox hazardous proportion analysis, using the SPSS version 11 program (SPSS Inc., Chicago, IL, USA). *P *< 0.05 was considered statistically significant.

## Results

### Expression of SDF-1/CXCR4 mRNA in cell lines and in human breast cancer tissures

**Figure 1:**
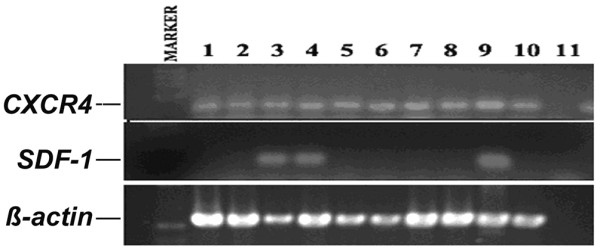
SDF-1/CXCR4 expression in various cell lines. 1: MDA-MB-157; 2: MDA-MB-231; 3: MDA-MB-435s; 4: MDA-MB-436; 5: MDA-MB-453; 6: MCF7; 7: BT549; 8: ZR751; 9: MRC5; 10: HECV; 11: negative control. CXCR, CXC chemokine receptor; SDF, stromal cell-derived factor.

SDF-1 mRNA was identified in MRC5, MDA-MB-435s, MDA-MB-436 and breast cancer tissues, but not in other breast cancer cell lines and HECV cells. It has been suggested that the MDA-MB-435 cell line is of melanocyte origin, and MDA-MB-436 was the only SDF-1 positive breast cancer cell line of all the lines tested in the present study. In contrast, CXCR4 mRNA expression was detected in all eight breast cancer cell lines, in MRC5 and HECV cells (Fig. 1), and in breast cancer tissue (data not shown). Quantitative analysis of the SDF-1 transcript revealed that breast tumour tissues had high levels of SDF-1 transcript (mean ± standard deviation: 195 ± 103 copies) as compared with normal mammary tissues (85.6 ± 54), but the difference was not statistically significant (*P *= 0.35). To take into account the contribution made by cellularity in mammary tissues, levels of SDF1 were normalized to the level of CK19. Dispite a higher SDF1:CK19 ratio in tumour tissues (39.3 ± 13.6) than in normal breast tissue (30.7 ± 3.97), the difference was not significant (*P *= 0.84). With respect to ER, those tumours negative for ER had higher levels of SDF-1 (246 ± 138) than did ER-positive tumours (57.9 ± 45.4; *P *= 0.20). A similar, insignificant trend was seen with ER-β (248.0 ± 131 for ER-β^- ^tumours and 1.3 ± 0.72 for ER-β^+ ^tumours). The SDF-1:CK19 ratio for ER-negative tumours was 52.7 ± 41.6 and that for ER-positive tumours was 30.8 ± 14.4 (*P *= 0.62). The ratio was 41.2 ± 17.3 for ER-β-negative and 8.3 ± 5.1 for ER-β-positive tumours (*P *= 0.072).

### SDF-1 has the potential to promote invasion and migration

**Figure 2:**
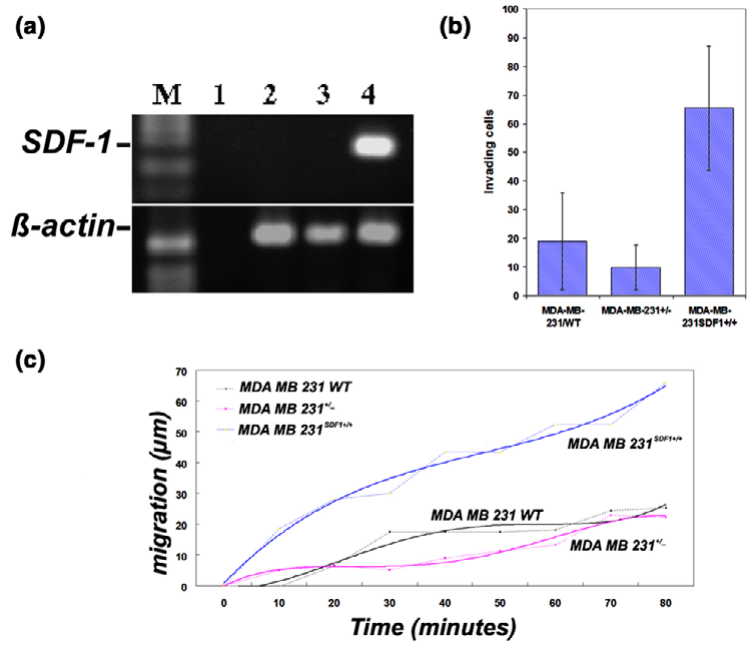
Manipulation of expression of SDF-1 in breast cancer cells. **(a) **The efficiency of stromal cell-derived factor (SDF)-1 transfected in MDA-MB-231 cells was confirmed by PCR. M: marker; 1: negative control; 2: MDA-MB-231 wild-type; 3: empty vector control MDA-MB-231^+/-^; 4: SDF-1-transfected MDA-MB-231SDF1^+/+^. **(b) **Invasiveness of transfected cells. *P < 0.01 versus control and wild-type. **(c) **Cellular migration.

MDA-MB-231SDF1^+/+ ^cells, which stably expressed SDF-1 (Fig. 2a), and MDA-MB-231^+/- ^(stable control plasmid transfectant) and wild-type MDA-MB-231 cells, which were SDF-1 negative, were tested for their invasiveness and migration. MDA-MB-231SDF1^+/+ ^cells exhibited greater invasiveness through Matrigel than did wild-type and MDA-MB-231^+/- ^cells (*P *< 0.01; Fig. 2b). In addition, the migration speed of MDA-MB-231SDF1^+/+ ^cells was markedly increased compared with the respective controls (Fig. 2c).

### SDF-1/CXCR4 immunohistochemical staining in human breast cancer

**Figure 3:**
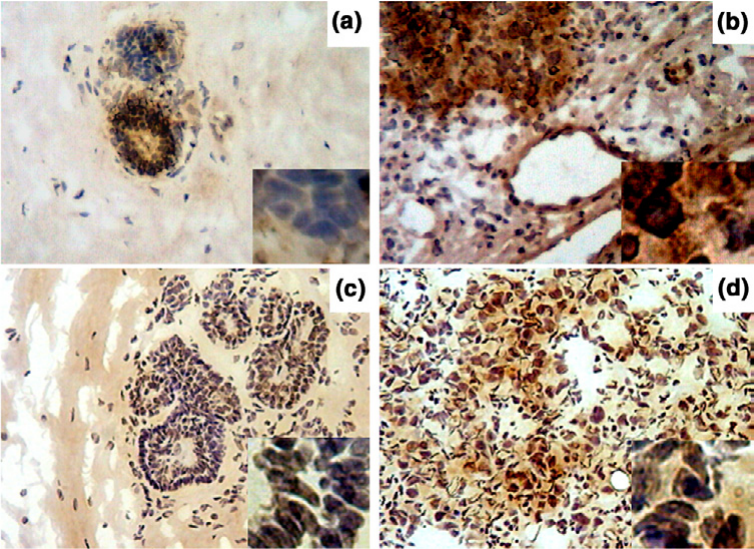
Immunohistochemical analysis of SDF1 and its receptor. Imunohistochemical staining of **(a,b) **SDF-1 and **(c,d) **the SDF-1 receptor CXCR4 in mammary tissues. The left panels show normal tissues, and the right panels show breast tumour tissues. CXCR, CXC chemokine receptor; SDF, stromal cell-derived factor.

Immunohistochemical staining confirmed expression of SDF-1 at the protein level in breast cancer tissue samples. In contrast to the adjacent nonmalignant tissue, we were able to demonstrate heterogeneous but consistent expression of SDF-1 antigen in tumour tissue. Immunohistochemical staining of SDF-1 appeared in most tumour cells and in stromal cells (Fig. 3a,b). As expected, staining of CXCR4 were seen in both normal and tumour cells (Fig. 3c,d), with staining in tumour cells being markedly stronger.

### SDF-1 expression and lymphatic nodal status, histological types, grades and staging

**Figure 4:**
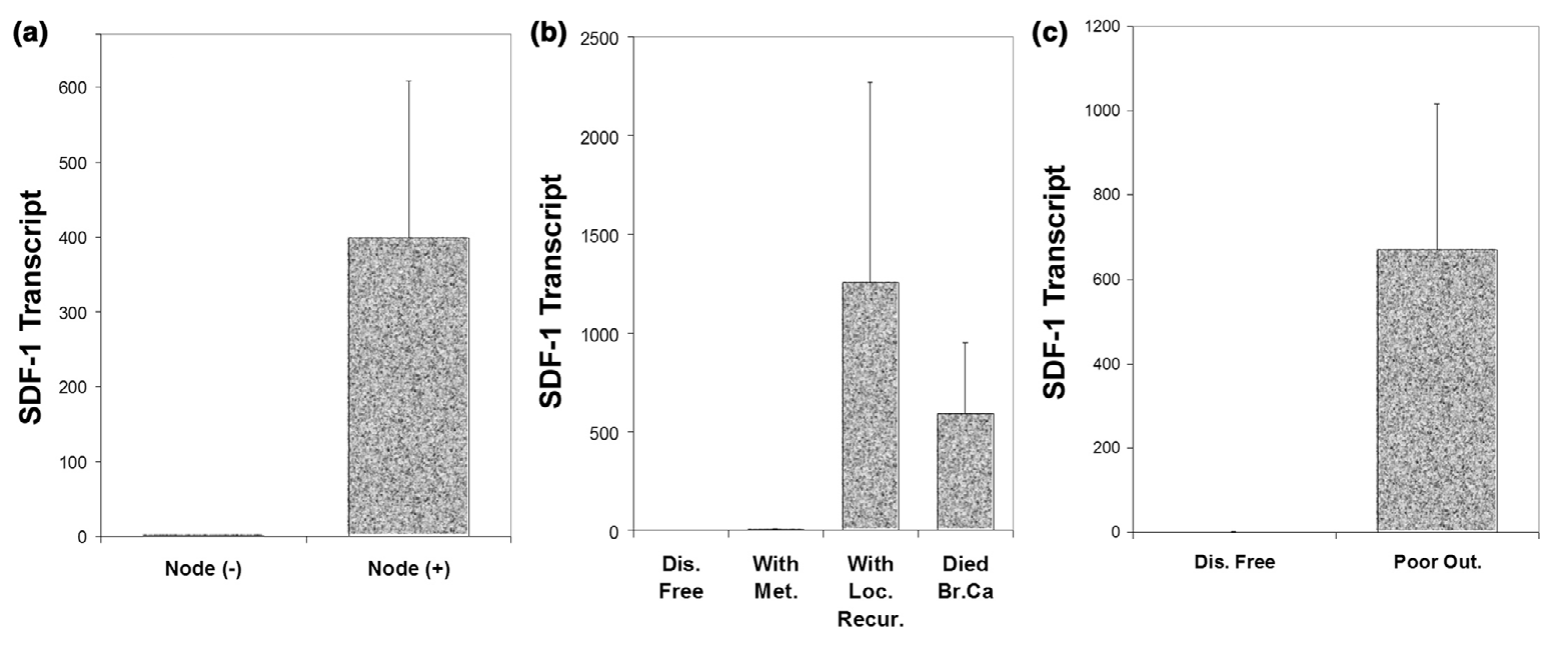
Levels of SDF-1 transcript in human breast tumours. **(a) **Stromal cell-derived factor (SDF)-1 expression level and lymph node metastasis, showing SDF-1 expression in node-negative and node-positive samples (0.89 ± 0.47 versus 399 ± 210; *P *= 0.05). **(b) **Significantly raised SDF-1 transcript in patients with local recurrence and with mortality. **(c) **Expression level of SDF-1 and clinical outcome (disease-free versus poor out come: 0.83 ± 0.35 versus 670 ± 346; *P *= 0.01).

We analyzed the levels of SDF-1 in relation to nodal status (Fig. 4a). Node-positive tumours had significantly higher levels of SDF-1 than did node-negative ones. The expression level of SDF-1 tended to be higher in the node-positive group, although there was no statistically significant difference between node-positive and node-negative groups (*P *= 0.05). The data were further analyzed by dividing node-positive and node-negative tumours into ER-positive and ER-negative groups. For SDF-1 no significant differences between subgroups were observed (ER^-^/node^- ^versus ER^-^/node^+^, *P *= 0.25; ER^-^/node^- ^versus ER^+^/node^-^, *P *= 0.57; P values for SDF1:CK19 were 0.27 and 0.32, respectively). No significant difference in SDF-1 was seen between ER-positive/node-negative and ER-positive/node-positive subgroups (*P *= 0.24; for SDF1:CK19 *P *= 0.27). Similarly, when node-positive and node-negative tumours were subdivided into ER-β-positive and ER-β-negative subgroups, no significant difference was seen.

We examined expression of SDF-1 relative to tumor types, grade and staging (Table 2). There was a trend in the differences in SDF1 expression between tumour grades, in that grade 3 and grade 2 tumours tended to have higher SDF1 levels than did grade 1 tumours, but this was not statistically significant. There were no significant relations between expression level of SDF-1 and tumor type and stage.

### SDF-1 expression correlated with prognosis and long term survival

The expression level of SDF-1 correlated with clinical outcome; patients with local recurrence (*P *= 0.05) and those who died from breast cancer (*P *= 0.03) had signfiantly higher levels of SDF-1 transcript (Fig. 4b). Those patients with metastasis and local recurrence, and who died from breast cancer had significantly higher levels of SDF-1 than did the disease-free group (*P *= 0.01; Fig. 4c).

**Figure 5:**
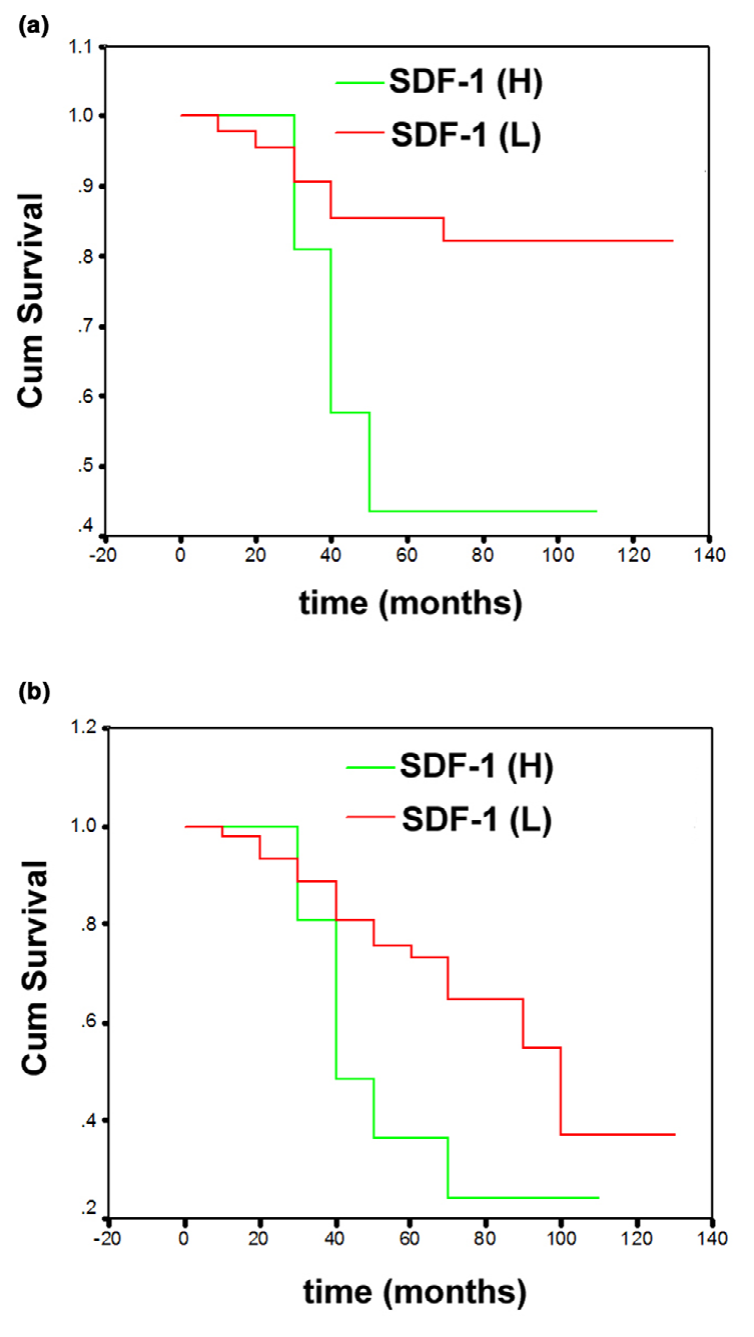
Kaplan–Meier survival curves. **(a) **Overall survival (*P *= 0.01). **(b) **Disease-free survival (*P *= 0.035). Median follow up: 72.2 months. Stromal cell-derived factor (SDF)-1 (H), patients with high levels of SDF-1 transcript (*n *= 79); SDF-1 (L), patients with low levels of SDF-1 transcript (*n *= 41).

To determine whether SDF-1 transcript levels were associated with long-term survival, we divided patients into those with high levels (*n *= 79) and those with low levels (*n *= 41) of SDF-1. The cutoff point was determined using the Nottingham Prognostic Index, and was set at the level at which patients had moderate prognoses (Nottingham Prognostic Index 3.4–5.4). As shown in the Kaplan–Meier survival curve (Fig. 5), high levels of SDF-1 significantly correlated with shorter overall survival (mean survival 94.1 months [95% confidence interval 65.4–122.9 months] versus 143.6 months [95% confidence interval 135.2–152.0 months] months for those with low levels of SDF-1; *P *= 0.001; Fig. 5a). Further analysis taking tumour grade into account was not possible because the sample number in each subgroup was too small. Similarly, high SDF-1 levels were associated with reduced incidence-free survival (*P *= 0.035 by Cox proportion analysis; Fig. 5b).

## Discussion

Chemokines are a family of small molecular weight proteins (8–10 kDa) that are classified into four distinct groups, depending on the positioning of the cysteine motif at the NH_2 _terminus. The family members include CXC, CC, C and CXXXC chemokines [26,27]. The specific effects of chemokines on their target cells are mediated by members of a family of seven-transmembrane-spanning, G-protein-coupled receptors [14,28].

SDF-1 is a member of the CXC subfamily of chemokines and its receptor is CXCR4. SDF-1 is constitutively expressed in various organs including bone, lung, liver, brain, thymus and lymph nodes [10,14,19], but SDF-1 is mainly produced by stromal cells, such as osteoblasts, fibroblasts and endothelial cells in the bone marrow [29,30]. Despite numerous studies on CXCR4 in breast cancer, reports on SDF-1 in human breast cancer are limited.

In the present study the expression of CXCR4 was detected in various cell lines and in malignant and nonmalignant breast tissues, but SDF-1 expression was only observed in two out of the eight breast cancer cell lines and in the fibroblast cell line MRC5. These results indicate that certain breast cancer cells co-express SDF-1 and CXCR4, which may act as a potential autocrine mechanism in breast cancer. We have reported that the fibroblast cell line, MRC5, strongly expressed SDF-1. Furthermore, in the present study immunohistochemical staining of SDF-1 was apparent in most tumour cells and in stromal cells. Collectively, from the results, we suggest that SDF-1 in breast cancer is produced by both tumour cells and stromal cells. The other potential source is the infiltrated immune cells, which frequently express CXCR4 and SDF1. The present study did not examine the proportion of these cells that produced SDF1 or the degree of expression, which would be an interesting focus for future studies.

The present study provides strong evidence that, when the SDF-1/CXCR4 complex existed (i.e. in MDA-MB-231SDF1^+/+ ^cells, which expressed both SDF-1 and CXCR4), breast cancer cells exhibited significant increases in invasiveness and faster migration. These findings suggest that breast cancer cells that co-express SDF-1 and CXCR4 may be more aggressive. In the present study we were unable to transfect fibroblasts with the current bacterial vector because no fibroblasts subsequently survived the electroporation and genetic marker selection process. It will be useful to develop viral expression for the purpose for future work. In addition, high levels of SDF-1 expression tended to be present in grade 3 and grade 2 tumors as compared with grade 1 tumours, further supporting the contention that breast cancer cells that express high levels of SDF-1 are more invasive.

Recently, studies implicated CXCR4 in chemotaxis, invasiveness and metastasis of tumours, particularly in metastasis of breast cancer, in an organ-specific manner. Muller and coworkers [19] found CXCR4 to be highly expressed in breast cancer cells, malignant breast tumours and metastases. On the other hand, peak levels of CXC chemokine ligand (CXCL)12 occurred in those organs that represent the initial destinations of breast cancer metastasis (i.e. lymph nodes, lung, liver and bone marrow). Furthermore, neutralizing the interaction between CXCL12 and CXCR4 significantly impaired metastasis to regional lymph nodes and lung in mice. Other reports have also shown that the SDF-1/CXCR4 biological axis is involved in regulating metastasis of tumours [6,18,31,32]. In the present study we found that that node-positive tumours had significantly higher levels of SDF-1 than did node-negative tumors, suggesting that SDF-1 may be involved in the lymph node metastatic process. Given that lymph node metastasis directly affects the prognosis of patients with breast cancer [4], we propose that SDF-1, via the CXCR4 pathway, is potentially a marker of nodal involvement. It was recently reported that SDF-1 can act as a direct target for ER-α in breast cancer cells (e.g. MCF-7 cells) [33,34]. In the present study it is noteworthy that EF-negative and ER-β-negative tumours tended to have higher levels of SDF-1. Although differences between these subgroups were not statistically significant, the trend, together with the *in vitro *studies, indicate that this link warrants further investigation. It is also noteworthy that SDF-1 expression in mammary tissues was primarily confined to stromal cells and, to some degree, cancer cells. We did not observe SDF-1 staining in vascular endothelial cells, HECV, and in vascular endothelial cells in the tissues – observations echoed by other studies [35,36]. This finding indicates that paracrine regulation may be the main pathway in breast cancer but that autocrine pathways may also exist. Secretion and production of SDF-1 are regulated by other factors. For example, expression of SDF-1 is decreased by IL-1, tumour necrosis factor and inflammation [37], whereas oestradiol can induce the production and secretion of SDF-1 in breast cancer cells [38]. On the other hand, tumour cells exposed to high concentrations of SDF-1 induce reduction in CXCR4 expression [18]. Furthermore, vascular edothelial grwoth factor can also induce CXCR4 expression in breast cancer cells [39,40]. Factors contributing to over-expression of SDF-1 in breast cancer thus warrant further investigation.

Finally, we demonstrated a significant correlation between SDF-1 expression and overall and disease-free survival in patients with breast cancer. The high level of SDF-1 expression suggests that there is a high likelihood of node metastasis, local recurrence and death from breast cancer in these patients. We and others found the expression pattern of CXCR4 to be significantly correlated with the degree of lymph node metastases but not with haematogenous metastases [41-43]. Therefore, SDF-1, together with its receptor CXCR4, may have potential value when assessing long-term clinical outcome in breast cancer.

## Conclusion

The present study demonstrated that breast cancer cells that express SDF-1, and therefore that have an active SDF-1/CXCR4 pathway, are more invasive and motile, thus have a more aggressive phenotype. In clinical breast cancers, and supported by data from cell lines, we found that SDF-1 appears to exist primarily in stromal cells and, to some degree, in breast cancer cells. That levels of SDF-1 are significantly correlated with nodal status, recurrence and, most notably, both overall and disease-free survival indicates that SDF-1 – and indeed the SDF-1 receptor complex – have strong predictive value in assessing long-term clinical outcome.

## Abbreviations

CXCL = CXC chemokine ligand; CXCR = CXC chemokine receptor; ER = oestrogen receptor; RT-PCR = reverse transcription polymerase chain reaction; SDF = Stromal cell derived factor.

## Authors' contributions

HK carried out *in vitro *testing and data analysis, and prepared the manuscript. GW conducted the immunohistochemistry study. CP contributed to the screening and ribozyme work. ADJ contributed to histological analysis. REM contributed to clinical follow ups. WGJ contributed to the study design, design of ribozymes, quantitative analysis of SDF1 transcript and statistical analysis.

## Figures and Tables

**Table 1:** Clinical features of patients included in the study

Clinical feature	*n*
Node status	
Node negative	65
Node positive	55
Grade	
1	23
2	41
3	56
Histology	
Ductal	88
Lobular	14
Others	8
TNM staging	
1	69
2	40
3	7
4	4
Clinical outcome	
Disease free	87
With metastasis	6
With local recurrence	5
Died from breast cancer	16
Died of unrelated disease	6

**Table 2:** SDF-1 expression and correlation with clinical pathology

Clinical pathology	SDF-1 level (mean ± SD)	*P*
Type		
Ductal	237 ± 131	
Lobular	88.9 ± 88.8	0.67
Others	0.89 ± 0.9	
Grade		
Grade 1	12.8 ± 12.5	
Grade 2	27 ± 27	0.07
Grade 3	371 ± 206	0.08
Staging		
TNM1	5.6 ± 4.5	
TNM2	290 ± 165	0.09
TNM3	1628 ± 1530	0.34
TNM4	1.1 ± 1.1	

SDF, stromal cell-derived factor.
